# Construction sites in Miami-Dade County, Florida are highly favorable environments for vector mosquitoes

**DOI:** 10.1371/journal.pone.0209625

**Published:** 2018-12-20

**Authors:** André B. B. Wilke, Chalmers Vasquez, William Petrie, Alberto J. Caban-Martinez, John C. Beier

**Affiliations:** 1 Department of Public Health Sciences, Miller School of Medicine, University of Miami, Miami, FL, United States of America; 2 Miami-Dade County Mosquito Control Division, Miami, FL, United States of America; University of Crete, GREECE

## Abstract

Urbanization is increasing globally, and construction sites are an integral part of the urbanization process. It is unknown to what extent construction sites create favorable breeding conditions for mosquitoes. The main objectives of the present study were to identify what species of mosquitoes are present at construction sites and the respective physical features associated with their production. Eleven construction sites were cross-sectionally surveyed for the presence of mosquitoes in Miami-Dade County, Florida including in areas previously affected by the Zika virus outbreak in 2016. A total of 3.351 mosquitoes were collected; 2.680 adults and 671 immatures. *Aedes aegypti* and *Culex quinquefasciatus* comprised 95% of all collected mosquitoes and were the only species found in their immature forms breeding inside construction sites. Results for the Shannon and Simpson indices, considering both immature and adult specimens, yielded the highest values for *Cx*. *quinquefasciatus* and *Ae*. *aegypti*. The individual rarefaction curves indicated that sampling sufficiency was highly asymptotic for *Cx*. *quinquefasciatus* and *Ae*. *aegypti*, and the plots of cumulative species abundance (ln S), Shannon index (H) and log evenness (ln E) (SHE) revealed the lack of heterogeneity of species composition, diversity and evenness for the mosquitoes found breeding in construction sites. The most productive construction site breeding features were elevator shafts, Jersey plastic barriers, flooded floors and stair shafts. The findings of this study indicate that vector mosquitoes breed in high numbers at construction sites and display reduced biodiversity comprising almost exclusively *Ae*. *aegypti* and *Cx*. *quinquefasciatus*. Such findings suggest that early phase construction sites have suitable conditions for the proliferation of vector mosquitoes. More studies are needed to identify modifiable worker- and organizational-level factors to improve mosquito control practices and guide future mosquito control strategies in urban environments.

## Background

Urbanization is increasing globally. It consists of altering the natural environment to accommodate the increasing human population, as well as people moving from rural areas to cities [1,2]. Such urbanization processes have been associated with the biotic homogenization of species [3], often leading to a decrease in the richness of species followed by an increase in the abundance of species capable of thriving in urban environments in a non-random process of biodiversity loss [4].

*Aedes aegypti* and *Culex quinquefasciatus* are among the mosquito species able to thrive in urban environments alongside the human population [5,6]. According to the Miami-Dade Mosquito Control Division, there are fifty mosquito species present in Miami, and in the last two years, during July to October, the most abundant species were *Culex nigripalpus* and *Aedes taeniorhynchus*. *Ae*. *aegypti* and *Cx*. *quinquefasciatus* represented around 30% of the total mosquitoes collected in Miami-Dade County between August 2016 and September 2018.

*Aedes aegypti* and *Cx*. *quinquefasciatus* can oviposit eggs in artificial breeding sites commonly found in urban environments where immature stages can thrive with virtually no predators and blood feed on vastly available human hosts [7]. Such conditions make it possible for these two species to rapidly invade and colonize new urban areas [8–12]. Furthermore, they can transmit many diseases. *Aedes aegypti* is the primary vector of dengue, chikungunya, yellow fever and Zika viruses [13–18], and *Cx*. *quinquefasciatus* is the primary vector of lymphatic filariasis, West Nile and Eastern Equine Encephalitis (EEE) viruses [19,20].

Miami-Dade County, Florida is at high risk of vector-borne disease outbreaks [21,22]. Its climate is conducive to the proliferation of mosquitoes and its unique geographical location is an entry port for the United States for many people coming from mosquito and arboviruses endemic areas [21,23,24].

Construction sites are an integral part of the urbanization process [25]. According to the Building Permits Survey from the United States Census Bureau 1,281,977 new permits for privately owned housing units have been issued in the United States in 2017 [26]. In 2015, the annual value of construction was $1.12 trillion, a 26% increase since 2010 after adjusting for inflation with expected increases in buildings due to urbanization [27]. Furthermore, Miami-Dade County is undergoing a rapid increase in urbanization. According to the Regulatory and Economic Resources Department of the Miami-Dade County, there were issued 138,000 building permits from 09/01/2016 to 08/30/2018, excluding renovations and additions [28]. Such a high number of construction sites may be translated to a substantial increase in the availability of breeding sites for vector mosquitoes [29].

Despite the importance of determining the role of construction sites in the production of vector mosquitoes, there is a paucity of rigorous studies on the subject. From the few studies that are available, it is unanimous that vector mosquitoes are successfully breeding in habitats present at construction sites across all life-cycles of the building (i.e., early, design, construction, operation, demolition and waste treatment) [29–33].

Based on initial observations by the Miami-Dade Mosquito Control Division, during the initial phases of construction, rainwater and groundwater can accumulate on elevator shafts, depressions on the floor, material piles and many other containers capable of holding water (S1 Fig), in which mosquitoes will have the opportunity to reproduce freely, without factors controlling their population expansion making it possible for them to reach high numbers in short periods of time [34,35]. Construction sites have also been correlated with the increase in the incidence of dengue in Brazil [36].

Therefore, this makes construction workers and the nearby community particularly vulnerable to bites of mosquitoes at construction sites. However, regardless of the epidemiological relevance of vector mosquitoes breeding at construction sites, there is a lack of awareness of both construction companies and workforce [31]. The risk they pose to the construction workforce and the general population is also unknown. Little is known about what mosquito species are present at construction sites, what features related to the construction sites affect and potentially drive their incidence and abundance, as well as to assess the inherent risks vector mosquitoes breeding in high numbers at construction sites represent to the local communities. The main objectives of the present study were to identify what species of mosquitoes are present at construction sites and the respective physical features associated with their production.

## Methods

### Study design

**Fig 1:**
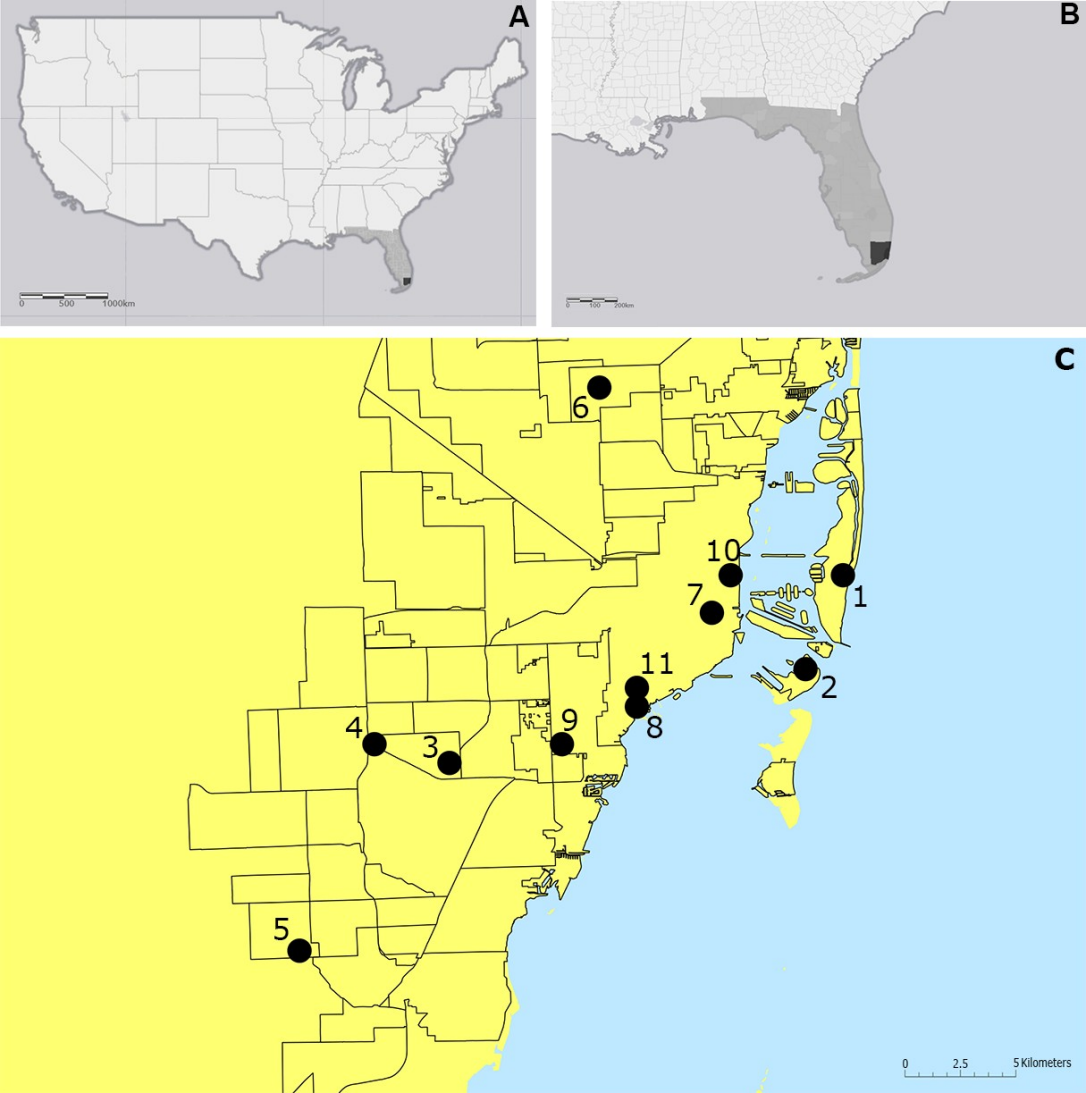
Maps displaying the surveyed construction sites in Miami-Dade County, Florida. (A) Continental United States; (B) Southeast United States; and (C) Miami-Dade County.

We used a cross-sectional study design to survey eleven construction sites in Miami-Dade County for immature and adult mosquitoes, including those geographic sites previously affected by the Zika virus outbreak in 2016 (Fig 1).

### Construction site recruitment/survey

Construction sites were chosen according to location, the phase of construction and size. The criteria for the selection of constructions sites was developed aiming to achieve comprehensive coverage of the County to assess the production of mosquitoes under different conditions and environments, comprising different neighborhoods with distinct socioeconomic and environmental characteristics. This sampling design allowed us to capture variation among nine different ZIP code regions and eleven neighborhoods located throughout Miami-Dade County, ranging from highly urbanized areas to suburban areas.

All construction sites were surveyed after the framing structure of the building was built entirely, but before doors and windows were in place sealing the building from outside elements. The size of construction sites was defined as: small, the size of a residential house or regular residential lot (300 m^2^); medium, commercial or residential building with no more than five stories or one acre; large, building with more than five stories or one acre (S1 Table).

### Mosquito collection approach

Collections were conducted in 2017 and 2018 during peak mosquito season (between July and October), and the sampling effort was standardized for all collections. Adult mosquitoes were collected with BG-Sentinel traps (Biogents AG, Regensburg, Germany) baited with dry ice [37], for 24 hours. The number of traps used was defined according to the size of each construction site: one BG-Sentinel trap was used to collect adult mosquitoes in small construction sites, four BG-Sentinel traps were used for medium size construction sites and five BG-Sentinel traps for large construction sites.

When possible, BG-Sentinel traps were deployed in similar locations among construction sites: (i) near the administration office or construction workers resting area; (ii) near Jersey plastic barriers; (iii) near elevator and stair shafts; and (iv) on upper floors near flooded areas.

Potential breeding sites were surveyed for immature mosquitoes. Larvae and pupae were collected with the aid of manual plastic pumps (turkey basters) and stored in plastic containers (100 ml) for transport. All the material collected was transported to the Miami-Dade County Mosquito Control Laboratory and subsequently morphologically identified using taxonomic keys [38].

Since this study poses less than minimal risk to participants and did not involve endangered or protected species the Institutional Review Board at the University of Miami determined that the study was exempt from institutional review board assessment (IRB Protocol Number: 20161212).

### Data analysis

Biodiversity indices were calculated for all collected mosquitoes based on the Shannon (H) and Simpson (1-D) indices [39,40]. These indices are commonly used together and are very useful to assess diversity patterns in a community. The Shannon index takes into consideration the number of specimens as well as the number of species, communities with fewer species yield values closer to 0 while communities with many species yield values closer to 1. The Simpson (1-D) index is used to assess evenness in a community, in which values closer to 0 means that species are equally present and values closer to 1 indicate the presence of more dominant species [41].

Individual rarefaction curves were generated to estimate both sampling sufficiency and the expected occurrence of species for smaller samples. This analysis is useful for comparing diversity in samples with different sizes, providing an estimation of the number of species in samples with fewer specimens. Plots of cumulative species log abundance (ln S), Shannon index (H) and log evenness (ln E) (SHE) profiles were also calculated for the collected mosquitoes. The ln S, H and ln E were calculated for the first sample, then the second sample was added to the model and indices were calculated again. This process was repeated for all samples generating a cumulative plot in which the changes in the direction of the lines indicate ecological heterogeneity of mosquito assembly [42].

A two-dimensional data matrix plot for mosquito relative abundance in construction sites was constructed using log_10_-transformed data in order to normalize the distribution [43]. Analyses were carried out with 10,000 randomizations without replacement and a 95% confidence interval using Past software (v.3.16) [44,45]. Fig 1 was produced using ArcGIS 10.2 (Esri, Redlands, CA).

## Results

Overall, 3,351 mosquitoes were collected at construction sites, including 2,680 adults (1,309 males and 1,371 females) and 671 immatures. The collected mosquitoes were distributed among five genera (*Aedes*, *Culex*, *Psorophora*, *Uranotaenia* and *Wyomyia*) and 13 species (*Aedes aegypti*, *Aedes albopictus*, *Ae*. *taeniorhynchus*, *Aedes tortilis*, *Aedes triseriatus*, *Culex coronator*, *Culex interrogator*, *Culex nigripalpus*, *Culex quinquefasciatus*, *Psorophora columbiae*, *Psorophora pygmea*, *Uranotaenia lowii*, *Wyomyia vanduzeei*) (Table 1).

**Table 1:** Total number of mosquitoes collected at construction sites in Miami-Dade County, Florida.

	Collection Site																						
Species	1		2		3		4		5		6		7		8		9		10		11		Total
	A	I	A	I	A	I	A	I	A	I	A	I	A	I	A	I	A	I	A	I	A	I	
*Aedes aegypti*	110								13		26		253	15 (3)		26 (42)	19	22 (12)	149	29 (52)	1096	26 (7)	1900
*Aedes albopictus*									2														2
*Aedes taeniorhynchus*	1		75																				76
*Aedes tortilis*	1		1		4																		6
*Aedes triseriatus*											1												1
*Culex coronator*											2												2
*Culex interrogator*											1												1
*Culex nigripalpus*			12						41		4												57
*Culex quinquefasciatus*	43		82				3		40		128		136	75 (4)		38 (3)	19	3	187	154 (2)	215	145 (13)	1290
*Psorophora columbiae*			1																				1
*Psorophora pygmaea*			11																				11
*Uranotaenia lowii*			2																				2
*Wyeomyia vanduzeei*									2														2

A = adults; I = immatures. In parentheses, number of pupae.

**Fig 2:**
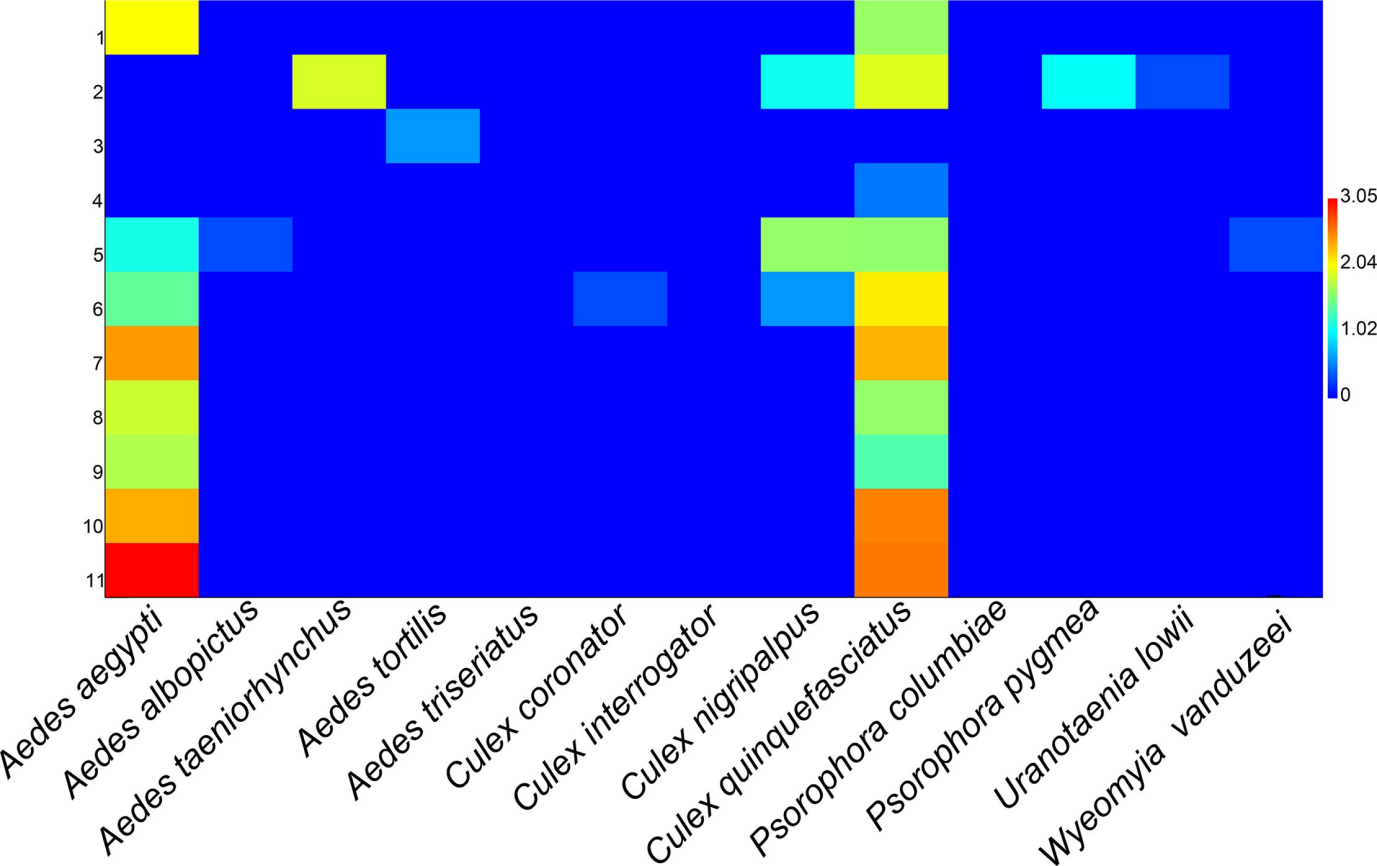
Two-dimensional data matrix plot for mosquito relative abundance by construction sites in Miami-Dade County, Florida. Y-axis = construction sites; X-axis = mosquito species. Mosquito relative abundance is represented by colors (blue = low abundance; red = high abundance).

*Aedes aegypti* and *Cx*. *quinquefasciatus* comprised 95% of all collected mosquitoes and were the only species found at construction sites in their immature form. *Aedes aegypti* was the most abundant mosquito collected at construction sites, comprising 1,900 specimens (1,666 adults and 234 immatures), followed by *Cx*. *quinquefasciatus* with 1,290 specimens collected (853 adults and 437 immatures) (Fig 2).

From the many potential breeding sites present at construction sites, such as stacks of construction supplies, empty soda cans, buckets, pipes, etc., the most productive breeding site features were elevator shafts, Jersey plastic barriers, flooded floors and stair shafts. Both *Ae*. *aegypti* and *Cx*. *quinquefasciatus* pupae were found in all breeding sites indicating that these habitats had suitable conditions for the development of mosquitoes. The most common breeding site for *Ae*. *aegypti* was Jersey plastic barriers totaling 124 specimens collected, comprising 54 larvae and 70 pupae.

On the other hand, the most common breeding site for *Cx*. *quinquefasciatus* was elevator shafts, in which 236 immature mosquitoes were collected, 233 larvae and 3 pupae. Moreover, *Cx*. *quinquefasciatus* was the most abundant mosquito species found in immature stages in a total of 437 specimens, 22 at the pupal stage. *Aedes aegypti*, from which from 234 specimens collected 116 were pupae (Table 2).

**Table 2:** Most common breeding sites for vector mosquitoes at construction sites in Miami-Dade County, Florida.

Breeding Site	*Aedes aegypti*	*Culex quinquefasciatus*	Total
Jersey Barrier	54 (70)	38 (3)	165
Elevator Shaft	27 (21)	233 (3)	284
Flooded Floor	25 (22)	75(4)	126
Stair Shaft	12 (3)	69(12)	96
Total	118 (116)	415 (22)	671

In parentheses, number of pupae.

**Fig 3:**
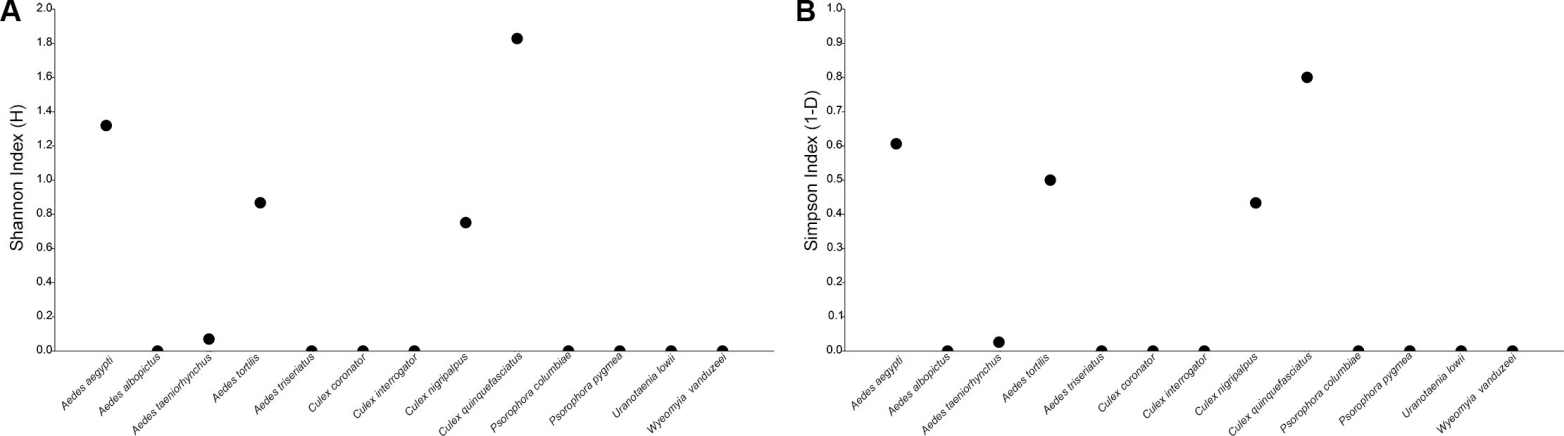
Shannon (A), and Simpson (B) indices for mosquitoes collected in construction sites in Miami-Dade County, Florida.

The Shannon’s diversity index averaged 0.372 (95% CI: 0.348–0.419). *Culex quinquefasciatus* yielded the highest values for the Shannon index 1.867 (95% CI: 1.828–1.781), followed by *Ae*. *aegypti* 1.319 (95% CI: 1.268–1.365). *Culex quinquefasciatus* was also the most dominant species based on the Simpson index (1-D) 0.800 (95% CI: 0.789–0.810), followed by *Ae*. *aegypti* 0.606 (95% CI: 0.583–0.627) (Fig 3).

**Fig 4:**
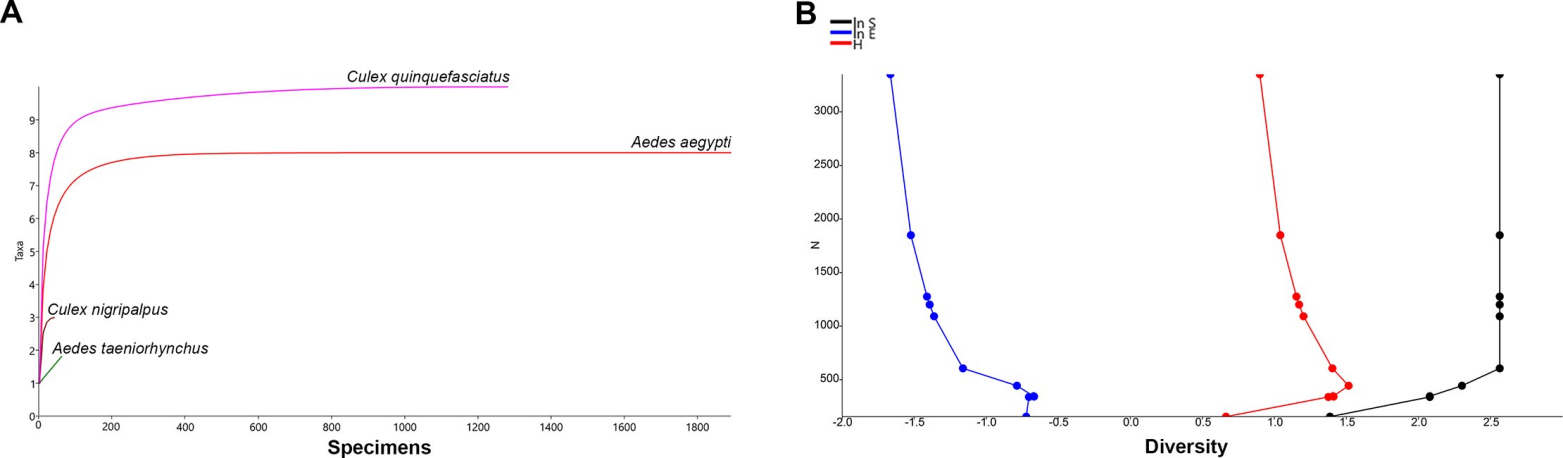
Biodiversity indices for mosquitoes collected in construction sites in Miami-Dade County, Florida. (A) Individual rarefaction curves (Y-axis = number of species; X-axis = number of specimens); (B) Plots of cumulative SHE profiles (ln S, H and ln E). (Y-axis = number of specimens; X-axis = diversity values for log abundance, Shannon index and log evenness).

The individual rarefaction curves indicated that sampling sufficiency was highly asymptotic for *Cx*. *quinquefasciatus* and *Ae*. *aegypti*, with a substantial degree of confidence for predicting the expected presence of those species for smaller sample sizes. The lack of substantial changes in the direction of the lines in the cumulative SHE analysis revealed the lack of heterogeneity of species composition, diversity and evenness for the mosquitoes found breeding in construction sites (Fig 4).

## Discussion

Recent outbreaks of Zika, dengue fever, chikungunya, yellow fever and West Nile viruses revealed that many countries are vulnerable to mosquito-borne pathogens transmitted mostly by *Aedes* and *Culex* mosquitoes, including the United States and many countries in Europe [17,21,22,46,47]. Our results indicate that construction sites have highly favorable conditions for vector mosquitoes in Miami-Dade County, Florida. The fact that *Ae*. *aegypti* and *Cx*. *quinquefasciatus* comprised the vast majority of collected mosquitoes (95%), and that they were the only species found in the immature stages, demonstrates that these two species are well adapted to thrive in the habitats present at construction sites. Immature mosquitoes were abundantly found at construction sites breeding in the accumulated stagnany water in elevator and stair shafts and Jersey plastic barriers (S2, S3 and S4 Figs).

Furthermore, despite the fact that collections were done in ecologically distinct areas, with different socioeconomic and environmental characteristics, the results were similar for all collections, in which *Cx*. *quinquefasciatus* and *Ae*. *aegypti* were the most dominant species. On the other hand, even though *Cx*. *nigripalpus* and *Ae*. *taeniorhynchus* are widely distributed throughout Miami and are more abundant than *Ae*. *aegypti* and *Cx*. *quinquefasciatus*, only a few specimens were found at construction sites as the ecological conditions of construction sites are not suitable for the proliferation of these species.

The results from the Shannon and Simpson indices were similar, with 4 from the 13 species found at construction sites yielding higher values, implying that construction sites have low biodiversity and few dominant species (i.e., *Cx*. *quinquefasciatus* and *Ae*. *aegypti*). These results are in agreement with the ones from the individual rarefaction curves, in which *Cx*. *quinquefasciatus* and *Ae*. *aegypti* were found to be highly likely to be found in smaller samples.

The lack of substantial variation in the SHE analysis indicated a low degree of heterogeneity in the composition of species at construction sites. The gradual decrease in the log Evenness (ln E) implied an unbalanced assembly of mosquitoes with two highly dominant species *Ae*. *aegypti* and *Cx*. *quinquefasciatus*. A similar result was found for the Shannon index (H), in which after an initial increase, its value gradually decreased due to the unbalance in the number of specimens collected for each species. These results were also reflected in the log abundance (ln S), in which the presence of highly dominant species resulted in the index values equilibrium with no further deviations.

The presence of specific features of construction sites may be associated with the high number of mosquitoes collected in this study. Construction sites are complex ever-changing environments. Medium and large enterprises often rely on having multiple phases of construction at the same time aiming for increasing the efficiency to complete the construction in a shorter time-span [48,49]. Such a chaotic and dynamic environment with an increased number of workers and materials at the job site create the appropriate conditions for vector mosquitoes [29,31,36]. Indeed, most mosquito control programs overlook the potential significance of construction sites both new construction and renovation projects. In the U.S., it is important to consider that the construction industry employs more than 9 million workers, representing 6% of the overall workforce [50].

Furthermore, construction workers are particularly vulnerable to bites of mosquitoes since they spend most of the day working outdoors. Outdoor workers, like those in construction, may be at increased risk of mosquito-borne diseases.

The infestation of the construction job site by vector mosquitoes stands as a significant challenge for public health, in which the ecology and behavior of mosquitoes as well as human behavior have to be taken into account. Changes in the phase of construction, human behavior or weather conditions may have significant influence in mosquito abundance. There is a critical need for the development of predictive models that assess for complex worker-mosquito interaction in the job site environment and inform mosquito control practices at the construction site.

Construction sites are particularly at higher risk for arbovirus transmission, as construction workers traveling from endemic regions can inadvertently become infected and then be subsequently bitten by vector mosquitoes that are being produced in high numbers at construction sites, possibly triggering an arbovirus outbreak. It is widely accepted that controlling the flow of infected people is virtually impossible, especially during the incubation period or in the case of asymptomatic cases [24,47,51]. Therefore, controlling mosquito populations is recognized as the most effective strategy for preventing vector-borne disease outbreaks [52,53].

The current safety guidelines for construction sites in the United States was issued in 2016 after the Zika virus outbreak by the Occupational Safety and Health Administration [54]. However, construction sites are complex and heterogeneous environments with several features accounting for the effectiveness of vector control guidelines, and, at that time, there was not enough information available about either how vector mosquitoes were able to exploit the construction sites habitats or how their ecology and behavior was being affected by such extreme conditions.

Safety guidelines for construction sites must account for the presence of vector mosquitoes and their interaction with construction workers and building features across the building cycle and with the different environments found at construction sites considering both space and time. Distinct vector control strategies may be required for different construction phases, since the efficiency of standard control strategies may be drastically reduced from one phase to another.

More studies are needed to address how workers and communities may be geographically and seasonally exposed to bites of vector mosquitoes, and to identify modifiable worker- and organizational-level factors that improve worksite mosquito control practices guide future mosquito control strategies in urban environments.

The findings of this study indicate that both immature and adult mosquitoes can be found in high numbers at construction sites and construction sites display reduced biodiversity of species sheltering almost exclusively *Ae*. *aegypti* and *Cx*. *quinquefasciatus*. Overall these findings suggest that construction sites have highly favorable environments for vector mosquitoes.

## Supporting information

S1 Table Information about the eleven surveyed construction sites in Miami-Dade County, Florida. Addresses contain only the street but not the complete address.
(DOCX)

S1 Fig Brochure developed by the Miami-Dade Mosquito Control division alerting for the risk of vector mosquitoes in construction sites. (TIF)

S2 Fig Example of a flooded elevator shaft inside a construction site. (A) Outside view of the elevator shaft; (B) rainwater accumulated on the bottom of the elevator shaft; and (C) stagnated water providing optimum conditions for the production of *Culex quinquefasciatus*.
(TIF)

S3 Fig Example of rainwater accumulated on the floor serving as breeding sites for *Aedes aegypti* mosquitoes. (A) Shallow pools of accumulated rainwater; (B) water with high contents of concrete dust and debris; and (C) immature specimens of *Aedes aegypti* breeding in this water collection.
(TIF)

S4 Fig Example of Jersey plastic barrier breeding vector mosquitoes. (A) Commonly found Jersey plastic barrier used in construction sites; (B) water accumulated on depression on top of Jersey barrier; (C) immature *Aedes aegypti* collected breeding in the accumulated water on top of Jersey barrier; and (D) adult *Aedes aegypti* inside main water reservoir of Jersey barrier.
(TIF)
