# Supplementary material for: Construction sites in Miami-Dade County, Florida are highly favorable environments for vector mosquitoes
Source: PLoS One. 2018 Dec 20;13(12):e0209625. doi: 10.1371/journal.pone.0209625 (PMC6301795; doi:10.1371/journal.pone.0209625)
Supplement: S1 Table — Addresses contain only the street but not the complete address. (DOCX) [file pone.0209625.s001.docx]

**Supplementary Table 1.** Information about the eleven surveyed construction sites in Miami-Dade County, Florida.

| **Collection Site** | **Description** | **Address** | **Zip code** | **Size** | **Collection Year** |
| --- | --- | --- | --- | --- | --- |
| 1 | Convention Center | Convention Center Dr, Miami Beach, FL | 33139 | Large | 2017 |
| 2 | Water Treatment Station | Rickenbacker Causeway, Key Biscayne, FL | 33149 | Medium | 2017 |
| 3 | Pump Station Renovation | Southwest 74th St, Miami, FL | 33173 | Small | 2017 |
| 4 | Pump Station Renovation | Southwest 116th Pl, Miami, FL | 33173 | Small | 2017 |
| 5 | Road and Sewage Renovation | Southwest 178th Terrace, Miami, FL | 33187 | Medium | 2017 |
| 6 | Warehouse | Northwest 37th Ave, Miami Gardens, FL | 33054 | Large | 2017 |
| 7 | Train Station | Northwest 1st Ave, Miami, FL | 33136 | Large | 2018 |
| 8 | Office Building | South Bayshore Dr, Miami, FL | 33133 | Medium | 2018 |
| 9 | Office Building | South Dixie Hwy, Coral Gables, FL | 33146 | Large | 2018 |
| 10 | Residential Building | Northeast 29th Terrace, Miami, FL | 33137 | Medium | 2018 |
| 11 | Garage Building | Southwest 28th Ln, Miami, FL | 33133 | Medium | 2018 |

Addresses contain only the street but not the complete address.
