# Supplementary figures and images for: Construction sites in Miami-Dade County, Florida are highly favorable environments for vector mosquitoes
Source: PLoS One. 2018 Dec 20;13(12):e0209625. doi: 10.1371/journal.pone.0209625 (PMC6301795; doi:10.1371/journal.pone.0209625)

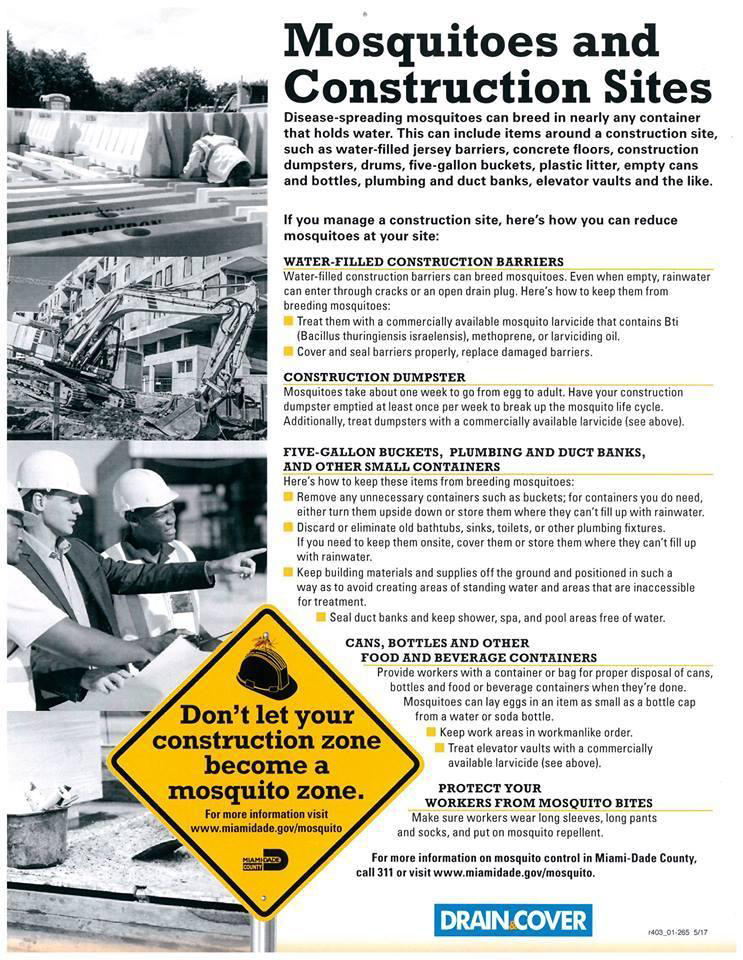

Supplement: S1 Fig — (TIF) [file pone.0209625.s002.tif]

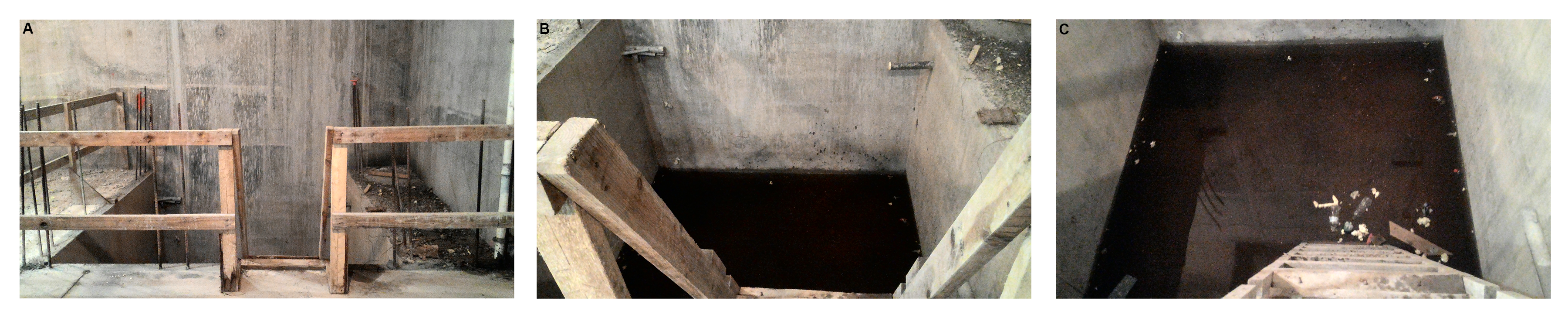

Supplement: S2 Fig — (A) Outside view of the elevator shaft; (B) rainwater accumulated on the bottom of the elevator shaft; and (C) stagnated water providing optimum conditions for the production of Culex quinquefasciatus. (TIF) [file pone.0209625.s003.tif]

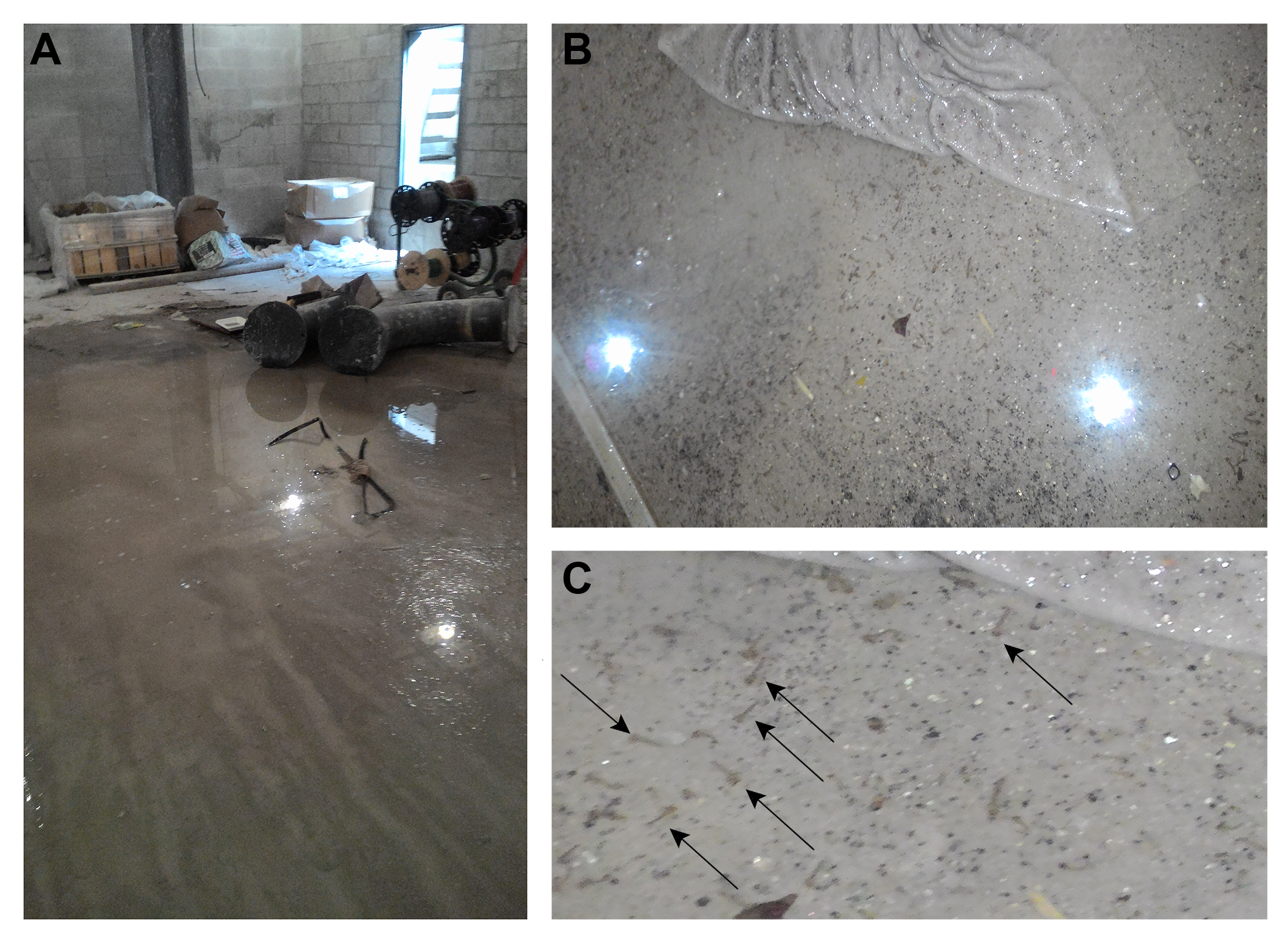

Supplement: S3 Fig — (A) Shallow pools of accumulated rainwater; (B) water with high contents of concrete dust and debris; and (C) immature specimens of Aedes aegypti breeding in this water collection. (TIF) [file pone.0209625.s004.tif]

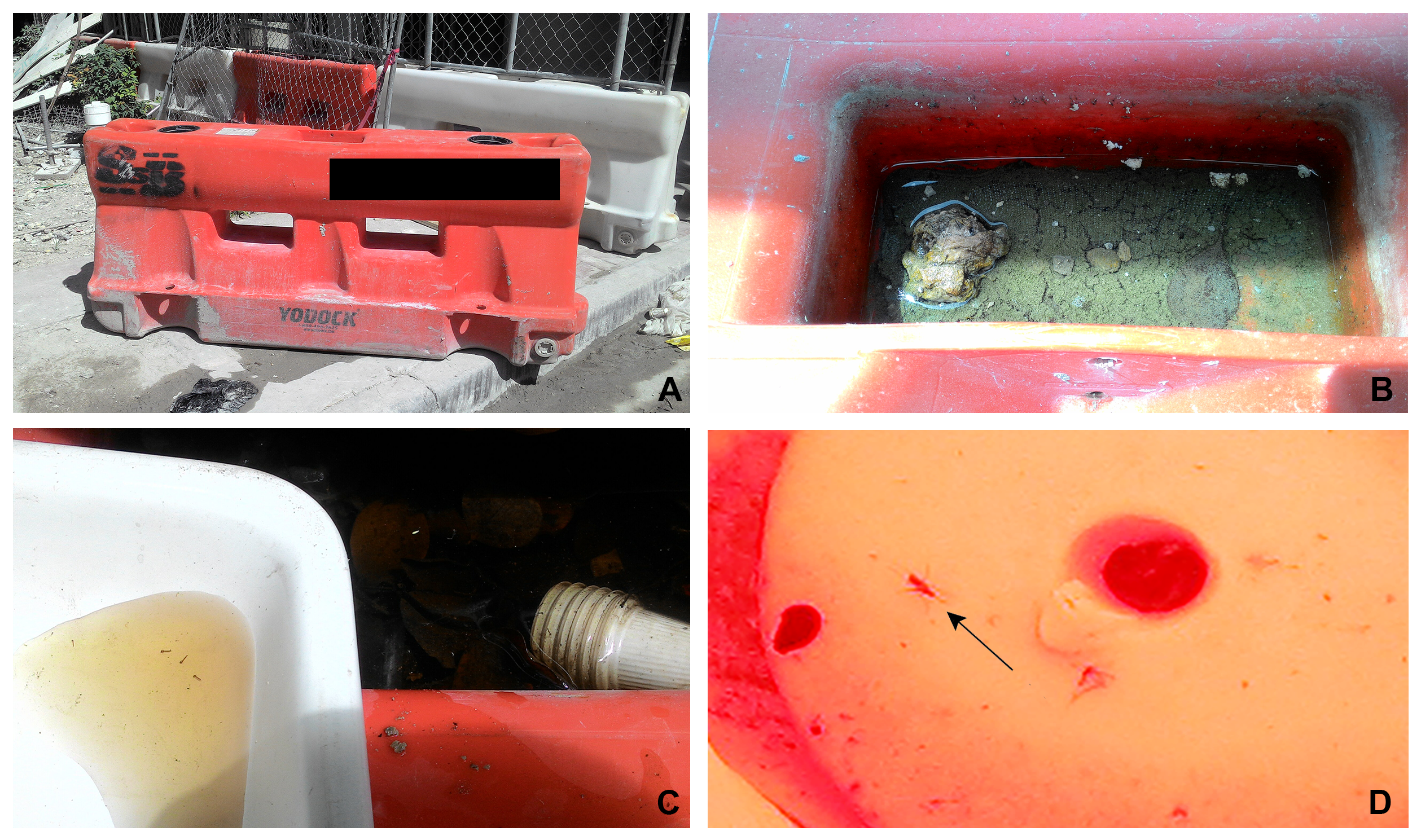

Supplement: S4 Fig — (A) Commonly found Jersey plastic barrier used in construction sites; (B) water accumulated on depression on top of Jersey barrier; (C) immature Aedes aegypti collected breeding in the accumulated water on top of Jersey barrier; and (D) adult Aedes aegypti inside main water reservoir of Jersey barrier. (TIF) [file pone.0209625.s005.tif]
